# Macroscopic Physical Properties and Nutrient Content in the Fermentation of Pangasius Waste by Single Cultures and Microbial Consortiums and Their Potential for Feed

**DOI:** 10.1002/mbo3.70040

**Published:** 2025-08-01

**Authors:** Abun Abun, Kiki Haetami, Denny Rusmana, Rahmad Fany Ramdhan

**Affiliations:** ^1^ Department of Nutrition and Animal Feed Technology Padjadjaran University Sumedang West Java Indonesia; ^2^ Department of Fisheries Padjadjaran University Sumedang West Java Indonesia

**Keywords:** chemical nutrient content, consortium method, fermentation, pangasius waste, physical macroscopic, single method

## Abstract

Fish waste processing through biological technology using fermentation microbes is an important concern in handling fishery waste, because fermentation can decompose organic waste materials into useful products and reduce pollutant levels. The purpose of the study was to select three types of microbes based on macroscopic properties and nutrient content of fermented pangasius waste (FPW). In the first stage, the fermentation of pangasius waste with eight species of microbes (three of bacteria, three of fungi, and two of yeast) is cultured in a single fermentation solid‐state medium. Each type of microbe that produces the highest number of colonies and nutrient content of FPW products in a single culture is then selected and used for consortium culture. The study used the nested, completely randomized design method, and the data were analyzed with ANOVA and Duncan's multiple range test. The results of single cultures of *Pseudomonas aeruginosa* (Pa), *Rhizopus microsporus* (Rm), and *Yarrowia lipolytica* (Yl) produced the best FPW products at 4 days of fermentation. The results of a consortium *P. aeruginosa*, *R. microsporus*, and *Y. lipolytica* (Pa + Rm + Yl) at a dose of 15% and fermentation for 4 days produced the best macroscopic physical properties, and its effect on nutrient content, obtained at a dose of 15% and fermentation for 4 days (protein 52.02%, fat 29.24%, and crude fiber 2.19%). The conclusion was that the consortium of three types of microbes (Pa + Rm + Yl) was more effective in treating FPW. FPW products are recommended as feed ingredients as a source of protein for poultry.

## Graphical abstract

This study is a new finding regarding the diversification of protein source raw material for livestock, namely, in the form of fermented pangasius waste. It is hoped that these findings can provide information regarding the use of protein source raw materials that are relatively more affordable and easy to obtain.

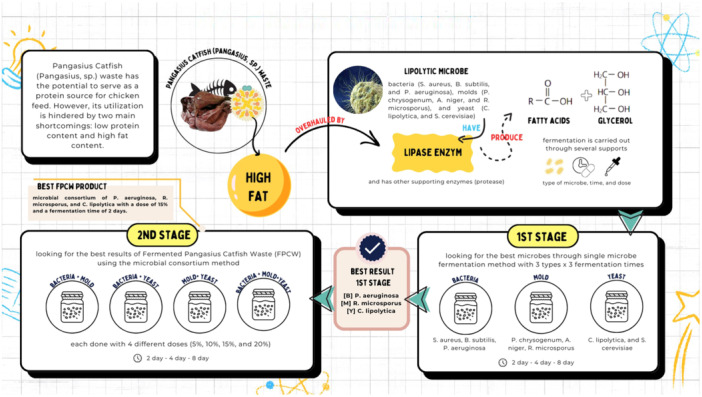

## 1 Introduction

Pangasius is an essential economic fish that generally lives in freshwater from the Indian subcontinent to the Indonesian archipelago. The genus pangasius in Indonesia is called pangasius spp, which has many species, such as Siamese pangasius (*Pangasianodon hypophthalmus*), jambal pangasius (*Pangasius djambal*), and pasupati pangasius (*Pangasius* sp.) (Sadi and Yoga 2021). Pangasius is a freshwater aquaculture fishery commodity that has a substantial market share both at home and abroad, one of which is used in the pangasius fillet industry (Palkar et al. 2017). The yield in the fish fillet processing process is around 45%; the remaining part (55%), including stomach contents, belly fat, bones, and pieces of skin, has not been utilized (Sankian et al. 2019).

Pangasius waste contains a lot of fat and is low in protein, especially in the head, belly meat, and stomach contents. The fat content ranged from 43.73% to 78.19% and protein 29.67% (Sankian et al. 2019). Lipid and protein components of fish waste are susceptible to degradation over time due to oxidative activity that promotes autolysis and the growth of harmful bacteria (Palkar et al. 2017). According to van't Land et al. (2017), the addition of 2.5% formic acid to fish waste to prevent the activity of decaying microbes, even though the preservative is used, there is still a damaging effect, especially on the protein fraction, thus affecting its stability, and the nutritional quality of fish silage decreases during storage.

Microbiological processing is one of the biological ways to improve the nutritional quality of fish waste (Marti‐Quijal et al. 2020). Fermentation technology can enhance the quality of nutrients, especially proteins and fats. In addition, fermentation technology can also preserve feed ingredients because it uses bacteria that, in the fermentation process, produce antimicrobial compounds, such as bacteriocin, which can inhibit the growth of putrefactive bacteria (Venegas‐Ortega et al. 2019).

Lipophilic compounds are essential in modern biotechnology, both in their use in living cells and as enzymes in biological processes (Johannah et al. 2018; Angelova and Schmauder 1999). So far, it is not widely known about the activity of microbes that act on fats (liphophilic), whether they can be lipolytic that can break down fats, in the application of fermentation of feed ingredients. The fermentation process includes the activities of amylase, protease, and lipase enzymes simultaneously with the determinants of fermentation success, namely, temperature, pH, oxygen, and microbes used (Toe et al. 2019; Abun et al. 2021).

In this study, the bacteria used were *Staphylococcus aureus*, *Bacillus subtilis*, and *Pseudomonas aeruginosa*, while the fungi types *Penicillium crisogenum*, *Aspergillus niger*, and *Rhizopus microsporus* were used, and the yeast types were *Yarrowia lipolytica* and *Saccharomyces cerevisiae*. Therefore, at all stages of fermentation, experiments are needed regarding selecting suitable microbes in pangasius waste to optimize microorganisms with the dosage and fermentation time so that quality products are obtained to meet the nutritional needs of livestock, especially poultry. It is crucial to determine microbial species of bacteria, fungi, and yeasts through a single‐microbial fermentation technique or a consortium to obtain fermented pangasius waste (FPW) so that it can be used as an alternative feed material for quality protein sources to meet livestock nutritional needs.

The purpose of the study is to determine species of lipophilic microbes, singly or in consortiums, by optimizing the inoculum dose and fermentation duration in improving the physical quality (macroscopic observation) and chemical quality (nutrient content) in FPW. It is believed that FPW products can be used as an alternative source of poultry feed protein.

## 2 Materials and Methods

Pangasius from the waste part is collected and then ground using a crushing mill machine, sterilized in an autoclave at a temperature of 121°C for 15 min, and stored in an airtight container.

The inoculum prepared includes primary inoculum culture and secondary inoculum culture according to the dose used. The manufacture of primary inoculum consists of three types of bacteria (*S. aureus*, *B. subtilis*, and *P. aeruginosa*), three types of fungi (*Pennicillium chrysogenum*, *A. niger*, and *R. microsporus*), and two types of yeast (*Y. lipolytica* and *S. cerevisiae*). Primary inoculum culture includes the preparation of a test tube container containing 10 mL of aqueous and microbial inoculums and a mixture of 90 mL of standard mineral solution consisting of 0.5% CO(NH_2_)_2_, 0.5% NaCl, 0.4% KH_2_ PO_4_, 0.1% MgCl_2_; fungi and yeast, 0.5% yeast extract, 0.5% CO(NH_2_)_2_, 0.05% KCl, 0.05% MgSO_4_, 0.001% CuSO_4_. To the mixture of inoculum and mineral solution, 1 g of pangasius waste and 1 g of nutrient broth are added. After that, the inoculum is incubated in an autoshaker bath at a temperature of 35°C for 8 days, and every 12 h, the total microbial colony (total plate count [TPC]) is counted.

The manufacture of secondary inoculum is carried out by dilution method of 10 mL of primary inoculum added with 90 mL of mineral solution. The mixture of primary inoculum and the mineral solution is then incubated as in the process of making primary inoculum for 8 days, and every 2 h, the TPC is calculated. After that, the secondary inoculum is incubated in an automatic shaker water bath at 35°C.

### 2.1 Fermented Pangasius Waste

Stage one: Sterile pangasius waste is inoculated with eight species of microorganisms, namely, bacteria (*S. aureus*, *B. subtilis*, and *P. aeruginosa*), fungi (*P. chrysogenum*, *A. niger*, *R. microsporus*), and yeast (*Y. lipolytica*, *S. cerevisiae*). *Sterile* pangasius waste is added: 1 mL of inoculum, nine mineral solutions, and 30 mL of aquades. Fermentation is anaerobically at 35°C for 2, 4, and 8 days.

The second stage is the propagation phase: Pangasius is fermented with a consortium consisting of the three best bacteria from the first selection of each type of microbe. The doses (secondary inoculum) used are 5%, 10%, 15%, and 20%, with a fermentation period of 2, 4, and 8 days. Each treatment was repeated three times. Fermentation is carried out anaerobic and solid‐state fermentation (SSF) at 35°C.

### 2.2 Physical (Macroscopic) and Chemical (Nutrient Content) Observations of FPW

Macroscopic physical observations include pH, temperature, total microbial colonies, and water content. pH is determined by dissolving the FPW sample in distilled water (1:2) and measured using a pH meter. The temperature is measured with a thermometer immersed in FPW fermentation media. The TPC was calculated by calculating the number of colonies in the FPW sample, and the moisture content was determined using the oven method.

Chemical observation of the nutritional content proximate includes crude protein, crude fiber, and crude fat from each treatment, with a method approved by the Association of Official Analytical Collaboration. Proximate analysis was carried out on samples with a fermentation time of 2, 4, and 8 days from each type of microbe. The AOAC method was used to determine the nutritional composition of proximates, namely, water content (protocol no. 925.10), protein (protocol no. 920.87), and fat (protocol no. 920.85) of FPW products.

### 2.3 Statistical Analysis

The experimental data were analyzed using a nested completely randomized design with analysis of variance and Duncan multiple range. The research design consists of two stages:

The first stage, the single‐microbial method, aims to obtain selected microbial types from bacteria, fungi, and yeasts. Two factors are included in each data analysis using a single culture method. The first factor is the type of species of each legal microbe (bacteria, fungi, and yeast), while the second factor is the fermentation time (2, 4, and 8 days), with three replicates.

The second stage aims to utilize selected microbes in a microbial consortium, two types of microbes and their mixtures, involving four combinations of (1) bacteria + fungi, (2) bacteria + yeast, (3) fungi + yeast, and (4) bacteria + fungi + yeast; with dosage factors (5%, 10%, 15%, and 20%), and fermentation time (2, 4, and 8 days), with three repetitions each.

## 3 Results

### 3.1 Results of Single Culture on Macroscopic Observation and Nutrient Content of FPW

The effect of microbial type and fermentation time on macroscopic observation and nutritional content of products FPW is presented in Tables 1 and 2. Table 1 shows that the pH of FPW did not have a significant effect (*p* > 0.05) is on microbial species, but was affected by fermentation time (*p* < 0.05). *P. aeruginosa* bacteria produced the lowest pH at 4 days (pH 5.10), fungi *R. microsporus* produced the lowest pH at 4 days (pH 5.13), and yeast *Yarrowia lipolytica* produced the lowest pH at 4 days (pH 5.18). The microbe type affects the FPW temperature increase (*p* < 0.05). At 4 days of fermentation, it was significantly higher (*p* < 0.05). The variance test showed that the use of microbial types had a significant (*p* < 0.05) effect on FPW temperature. In terms of microbial type, it affected temperature, with the highest temperature in *P. aeruginosa* bacteria at 4 days of fermentation (34.97°C). The highest temperature in *R. microsporus* fungi was at 4 days of fermentation (32.75°C), and the highest in *Y. lipolytica* yeast was at 4 days of fermentation (34.45°C). The moisture content of FPW was not significant (*p* > 0.05) and was influenced by the bacterial species, but the fermentation time had a significant effect (*p* < 0.05) on FPW. The highest moisture content was in *P. aeruginosa* bacteria with a fermentation time of 4 days (73.57%), in fungi, namely, *R. microsporus* with a fermentation time of 4 days (73.15%), and in *Y. lipolytica* yeast with a fermentation time of 4 days (72.83%). The number of FPW bacterial colonies was not significant (*p* > 0.05) affected by microbial species but was influenced by fermentation time (*p* < 0.05). *P. aeruginosa* bacteria produced the highest TPC at 4 days (156 × 10^9^ cfu/mL). Table 1 shows that the best macroscopic observations are in *P. aeruginosa* bacteria, *R. microsporus* fungi, and *Y. lipolytica* yeast, with a fermentation time of 4 days.

**Table 1:** Macroscopic observation of FPW at different times of fermentation using a single microbe.

Microbe	Fermentation time (day)	pH	Temperature (°C)	Moisture (%)	TPC (×10^9^ cfu/mL)
Bacteria					
*Staphylococcus aureus*	2	5.74^b^	28.40^a^	69.29^b^	63^b^
	4	5.40^ab^	33.40^cd^	70.17^b^	101^c^
	8	6.37^c^	30.20^b^	62.90^a^	94^b^ ^c^
*Bacillus subtilis*	2	5.82^bc^	29.10^a^	69.92^b^	28^a^
	4	5.17^ab^	32.00^c^	71.21^bc^	124^cd^
	8	6.74^c^	28.60^a^	68.22^b^	64^b^
*Pseudomonas aeruginosa*	2	5.75^b^	29.90^a^	68.98^b^	47^ab^
	4	5.10^a^	34.97^d^	73.57^c^	156^d^
	8	6.80^c^	32.30^c^	62.70^a^	91^bc^
Fungi					
*Pennicillium chrysogenum*	2	5.93^bc^	29.30^a^	72.37^c^	43^ab^
	4	5.27^ab^	32.00^c^	72.81^c^	62^b^
	8	6.23^c^	31.57^bc^	58.53^a^	38^a^
*Aspergillus niger*	2	5.83^b^	30.60^b^	72.57^c^	40^ab^
	4	5.16^ab^	32.53^c^	72.23^c^	64^b^
	8	6.70^c^	30.33^b^	62.18^ab^	42^ab^
*Rhizopus microsporus*	2	5.31^ab^	28.63a	70.21^b^	28^a^
	4	5.13^a^	32.75^cd^	73.15^c^	146^d^
	8	6.63^c^	31.33^bc^	72.34^c^	90b^c^
Yeast					
*Saccharomyces cerevisiae*	2	5.76^b^	30.90^b^	71.72^bc^	25^a^
	4	5.26^ab^	34.17^d^	71.24^bc^	60^b^
	8	6.59^c^	31.47^bc^	70.15^b^	57^ab^
*Yarrowia lipolytica*	2	5.83^bc^	31.02^bc^	67.35^ab^	42^ab^
	4	5.18^ab^	34.45^d^	72.83^c^	93^cd^
	8	6.63^c^	32.25^c^	63.68^ab^	85^bc^

*Note:* ^a–d^Means with different superscripts in the same column differ significantly (*p* < 0.05).

Abbreviations: FPW, fermented pangasius waste; TPC, total plate count.

**Table 2:** Nutrient content (crude protein, crude fiber, and crude fat) FPW and moisture of FPW drying results using a single microbe.

Bacteria	Fermentation time (day)	Crude protein (%)	Crude fiber (%)	Crude fat (%)	Moisture (%)
Bacteria					
*Staphylococcus aureus*	2	35.12^b^	2.85^c^	41.58^b^	10.00^c^
	4	34.43^b^	2.70^b^	45.82^c^	11.50^cd^
	8	30.95^a^	2.68^b^	49.16^d^	13.33^d^
*Bacillus subtilis*	2	40.18^c^	2.98^c^	41.23^b^	8.93^ab^
	4	38.15^bc^	2.83^c^	44.52^c^	9.17^b^
	8	36.88^b^	2.72^b^	50.59^d^	10.00^b^
*Pseudomonas aeruginosa*	2	40.80^c^	2.82^c^	36.50^a^	8.33^a^
	4	38.44^bc^	2.42^a^	46.27^c^	9.33^b^
	8	32.67^ab^	2.30^a^	51.39^d^	10.17^c^
Fungi					
*Pennicillium chrysogenum*	2	36.59^b^	2.95^c^	40.59^b^	11.57^cd^
	4	33.76^ab^	2.80^c^	45.19^c^	12.13^cd^
	8	33.22^ab^	2.62^b^	50.51^d^	13.87^d^
*Aspergillus niger*	2	38.20^bc^	2.98^c^	43.33^bc^	9.23^b^
	4	32.69^ab^	2.84^c^	45.81^c^	10.67^bc^
	8	30.53^a^	2.69^b^	53.48^d^	11.03^bc^
*Rhizopus microsporus*	2	39.77^c^	2.70^b^	38.39^ab^	8.97^ab^
	4	36.87^b^	2.57^ab^	38.83^ab^	10.11^ab^
	8	31.78^a^	2.50^a^	51.73^d^	11.24^bc^
Yeast					
*Saccharomyces cerevisiae*	2	35.54^b^	2.80^c^	40.84^b^	12.24^cd^
	4	32.37^ab^	2.60^b^	43.97^bc^	12.78^cd^
	8	29.48^a^	2.50^ab^	49.69^d^	13.22d
*Yarrowia lipolytica*	2	38.69^bc^	2.79^c^	39.32^ab^	10.15^ab^
	4	32.86^ab^	2.55^ab^	45.57^c^	11.23^bc^
	8	31.88^a^	2.41^a^	51.14^d^	12.45^cd^

*Note:* ^a–d^Means with different superscripts in the same column differ significantly (*p* < 0.05).

Abbreviation: FPW, fermented pangasius waste.

Table 2 provides an overview of the nutritional value of FPW, which, in general, is affected by the type of microbes and the duration of fermentation. The variance test showed that the use of microbes had a significant effect (*p* < 0.05) on the crude protein content of FPW. *P. aeruginosa* bacteria, at a fermentation time of 2 days, produced the highest crude protein content (40.80%). As for *R. microsporus* fungi at 2 days of fermentation, it produces a crude protein content of 39.77%, and yeast *Y. lipolytica* produces a crude protein content of 38.69%. The crude fiber content of FPW was not significant (*p* > 0.05) and was influenced by microbial species but by fermentation time. The variance test showed that the fermentation time had a significant effect (*p* < 0.05) on the crude fiber content of FPW. The lowest crude fiber content is produced by *P. aeruginosa* bacteria, which is 2.42% at 4 days of fermentation. The fungi *R. microsporus*, which is 2.57% at the fermentation time of 4 days, and yeast *Y. lipolytica*, which is 2.55% at the fermentation time of 4 days. The crude fat content of FPW was not significantly (*p* > 0.05) influenced by microbial species but was influenced by fermentation time (*p* < 0.05). The lowest crude fat content was in *P. aeruginosa* bacteria with a fermentation time of 2 days (36.50%), *fungi R. microsporus* with a fermentation time of 2 days (38.39%), and yeast *Y. lipolytica* with a fermentation time of 2 days (39.32%). The moisture content of FPW (after drying) was not noticeable (*p* > 0.05) and was influenced by microbial species, but significant (*p* > 0.05) was affected by fermentation time (*p* < 0.05). The lowest moisture content was in *P. aeruginosa* bacteria with a fermentation time of 2 days (8.33%), *R. microsporus* fungi with a fermentation time of 2 days (8.97%), and *Y. lipolytica* yeast with a fermentation time of 2 days (10.15%). Table 2 provides an overview of the best nutrient content in FPW, using *P. aeruginosa* bacteria, *R. microsporus* fungi, and *Y. lipolytica* yeast, with a fermentation time of 2 days.

### 3.2 Results of Consortium Culture on Macroscopic Observation and Nutrient Content of FPW

The results of the selection in the first stage obtained the best three species from each type of microbe, namely, *P. aeruginosa* (Pa) bacteria, *R. microsporus* (Rm) fungi, and *Y. lipolytyca* (Yl) yeast, and can live synergistically in pangasius waste media with high protein and fat content. The results of statistical analysis (Table 3) showed that fermentation at a dose of 15% and a fermentation time of 4 days significantly (*p* < 0.05) lowered the pH value, with the lowest pH obtained in the Pa + Rm + Yl consortium (4.88). Each type of consortium microbes had a significant influence (*p* < 0.05) on the increase in temperature, moisture content, and total PWF microbial colonies, along with fermentation time. The highest temperature, moisture content, and total microbial colonies were found in FPW, which used a consortium of Pa + Rm + Yl microbes at a 15% inoculum dose with a fermentation time of 4 days, namely, a temperature of 35.40°C, a moisture content of 72.54%, and a TPC of 216 × 10^9^ cfu/mL.

**Table 3:** Macroscopic observation of FPW for consortium selection using a consortium of microbes.

Consortium microbe	Dose consortium (%)	Fermentation time (day)	pH	Temperature (°C)	Moisture (%)	TPC (×10^9^ cfu/mL)
Pa + Rm	5	2	5.98^b^	28.72^a^	67.13^b^	58.67^bc^
		4	6.10^b^	29.87^a^	68.54^bc^	61.77^c^
		8	6.74^d^	31.28^b^	69.15^c^	72.35^c^
	10	2	6.16^b^	28.42^a^	64.60^b^	68.00^c^
		4	6.32^c^	29.87^a^	66.44^b^	87.00^c^
		8	6.48^d^	31.18^b^	67.75^b^	90.67^c^
	15	2	5.49^ab^	28.59^a^	66.42^b^	84.55^c^
		4	5.55^ab^	33.30^cd^	72.03^cd^	131.37^cd^
		8	6.80^d^	32.47^c^	71.20^c^	126.54^cd^
	20	2	6.07^b^	27.80^a^	66.89^b^	99.45^cd^
		4	6.31^c^	30.77^ab^	69.55^c^	125.87^cd^
		8	6.50^d^	31.84^b^	69.50^c^	133.67^cd^
Pa + Yl	5	2	5.63^b^	29.28^a^	68.50^bc^	31.28^a^
		4	6.03^b^	30.87^a^	69.80^c^	46.76^b^
		8	5.84^b^	31.42^b^	70.25^c^	68.45^c^
	10	2	5.73^b^	29.55^a^	70.95^c^	26.93^a^
		4	6.07^b^	29.87^a^	69.75^c^	87.75^c^
		8	5.93^b^	30.38^ab^	71.50^cd^	94.58^cd^
	15	2	5.81^b^	29.45^a^	69.00^c^	94.83^cd^
		4	5.58^ab^	33.13^cd^	72.50^d^	139.53^d^
		8	6.89^d^	32.90^c^	71.35^cd^	101.21^cd^
	20	2	5.80^b^	28.90^a^	67.55^b^	45.55^b^
		4	6.36^c^	30.77^a^	72.00^d^	77.57^c^
		8	6.51^d^	31.17^b^	70.90^c^	87.56^c^
Rm + Yl	5	2	5.92^b^	30.87^a^	66.42^b^	36.29^b^
		4	5.60^b^	31.55^b^	67.48^b^	58.34^c^
		8	5.76^b^	31.47^b^	69.68^c^	69.23^c^
	10	2	5.73^b^	30.09^a^	62.97^a^	52.25^b^
		4	6.19^b^	32.15^c^	67.13^b^	66.33^c^
		8	6.34^c^	32.07^c^	70.55^c^	74.79^c^
	15	2	5.86^b^	30.60^a^	69.04^c^	69.98^c^
		4	5.51^ab^	33.73^cd^	71.95^cd^	74.78^c^
		8	6.03^b^	32.73^c^	71.75^cd^	80.99^c^
	20	2	5.74^b^	29.47^a^	70.52^c^	60.82^c^
		4	5.54^b^	32.07^c^	71.52^cd^	69.23^c^
		8	5.63^b^	31.99^b^	70.83^c^	78.66^c^
Pa + Rm + Yl	5	2	6.60^d^	30.37^a^	66.21^b^	23.67^a^
		4	6.30^c^	31.67^b^	67.12^b^	40.00^b^
		8	5.75^b^	32.32^c^	70.94^c^	49.27^b^
	10	2	5.65^b^	30.75^a^	62.54^a^	35.67^b^
		4	5.76^b^	32.87^c^	67.21^b^	42.00^b^
		8	6.51^cd^	32.69^c^	71.40^cd^	43.23^b^
	15	2	5.98^b^	30.98^a^	69.96^c^	154.50^d^
		4	4.88^a^	35.40^d^	72.54^d^	216.00^e^
		8	5.70^b^	33.42^cd^	72.30^d^	162.67^d^
	20	2	5.70^b^	30.80^a^	65.70^b^	54.00^c^
		4	5.78^b^	31.70^b^	67.53^b^	67.67^c^
		8	5.79^b^	33.98^cd^	69.83^c^	96.00^cd^

*Note:* ^a–d^Means with different superscripts in the same column differ significantly (*p* < 0.05).

Abbreviations: FPW, fermented pangasius waste; TPC, total plate count.

The influence of microbial consortiums on nutrient content is presented in Table 4. The results of the variance test showed that the use of the dose and fermentation time of the microbial consortium had a significant influence (*p* < 0.05) on the content of crude protein, crude fiber, crude fat, and moisture content of FPW products (after drying). On the basis of Table 4, the highest protein content and the lowest content of crude fiber, crude fat, and moisture content were found in FPW using a microbial consortium of Pa + Rm + Yl at a dose of 15% for 4 days, namely, protein content of 52.02%, crude fiber 2.19%, crude fat 29.24%, and water content of 8.82%. FPW products by a consortium of three types of microbes (*P. aeruginosa*, *R. microsporus*, and *Y. lipolytica*) are suitable as feed sources for protein for poultry nutrition.

**Table 4:** Nutrient content (crude protein, crude fiber, and crude fat) FPW and moisture of FPW drying results using microbial consortium.

Consortium microbe	Dose consortium (%)	Fermentation time (day)	Crude protein (%)	Crude fiber (%)	Crude fat (%)	Moisture (%)
Pa + Rm	5	2	32.37^a^	3.39^cd^	53.56^d^	12.12^bc^
		4	36.53^a^	3.25^cd^	44.86^b^	11.15^b^
		8	41.55^b^	2.78^b^	42.50^b^	11.31^b^
	10	2	39.01^b^	4.05^d^	53.14^d^	11.20^b^
		4	45.15^c^	2.75^b^	47.65^bc^	10.00^ab^
		8	47.20^c^	2.69^b^	48.83^bc^	10.28^ab^
	15	2	40.58^b^	3.27^cd^	53.36^d^	10.19^ab^
		4	49.71^d^	2.89^b^	42.75^b^	9.24^ab^
		8	48.95^d^	2.35^ab^	38.26^ab^	8.89^a^
	20	2	48.10^d^	3.42^cd^	48.02^bc^	10.34^ab^
		4	49.55^d^	3.01^c^	44.96^b^	11.30^b^
		8	42.31^c^	2.72^b^	41.12^b^	10.26^ab^
Pa + Yl	5	2	30.41^a^	3.77^d^	59.09^e^	13.44^d^
		4	32.11^a^	3.45^cd^	47.16^bc^	12.49^c^
		8	36.35^a^	2.44^ab^	37.81^ab^	11.74^bc^
	10	2	34.95^a^	3.78^d^	55.52^d^	11.43^b^
		4	36.76^a^	3.75^d^	54.82^d^	10.98^b^
		8	40.31^b^	2.83^b^	38.03^ab^	10.21^ab^
	15	2	36.64^a^	2.66^b^	46.00^bc^	9.83^ab^
		4	50.62^de^	2.41^ab^	37.28^ab^	8.91^a^
		8	44.42^c^	2.56^ab^	41.62^b^	9.14^ab^
	20	2	43.12^c^	2.74^b^	51.67^c^	11.89^bc^
		4	47.73^c^	2.59^ab^	41.42^b^	10.84^b^
		8	44.35^c^	2.56^ab^	43.69^b^	10.41^ab^
Rm + Yl	5	2	35.84^a^	3.34^cd^	42.16^b^	10.01^ab^
		4	39.04^b^	3.47^cd^	39.53^b^	11.32^b^
		8	40.10^b^	3.17^c^	39.26^b^	12.48^c^
	10	2	36.36^ab^	3.24^cd^	48.60^c^	10.79^ab^
		4	40.79^b^	3.05^c^	43.90^b^	12.28^c^
		8	50.23^de^	2.76^b^	42.49^b^	11.81^bc^
	15	2	37.69^b^	2.81^b^	43.65^b^	9.20^ab^
		4	50.25^de^	2.42^ab^	36.62^ab^	8.84^a^
		8	41.43^c^	2.41^ab^	35.80^ab^	9.13^ab^
	20	2	37.72^b^	27.97^b^	45.98^bc^	11.03^b^
		4	43.98^c^	26.12^b^	41.19^b^	12.10^ab^
		8	50.05^de^	25.32^ab^	39.26^b^	12.88^d^
Pa + Rm + Yl	5	2	35.84^a^	3.28^c^	46.38^bc^	10.58^ab^
		4	38.53^b^	2.86^b^	55.89^d^	11.66^bc^
		8	42.22^c^	2.71^b^	55.17^d^	12.36^c^
	10	2	47.04^c^	3.40^c^	42.46^b^	10.46^ab^
		4	49.15^d^	2.76^b^	52.53^cd^	12.38^c^
		8	50.55^de^	2.64^b^	48.02^bc^	11.27^b^
	15	2	48.03^d^	2.82^b^	45.65^b^	9.14^ab^
		4	52.02^e^	2.19^a^	29.24^a^	8.82^a^
		8	52.79^e^	2.37^ab^	56.42^d^	9.04^a^
	20	2	46.29^c^	2.82^b^	46.71^bc^	10.24^ab^
		4	48.83^d^	2.46^ab^	57.53^d^	11.64^bc^
		8	50.07^de^	2.43^ab^	57.80^d^	13.31^d^

*Note:* ^a–e^Means with different superscripts in the same column differ significantly (*p* < 0.05).

Abbreviation: FPW, fermented pangasius waste.

## 4 Discussion

### 4.1 Effect of the Use of a Single Microbe on the Physical and Chemical Quality of FPW

#### 4.1.1 Macroscopic Observations

Microbial activity in the fermentation process significantly decreased the pH value of FPW, with the lowest pH in *P. aeruginosa* bacteria (5.1), *R. microsporus* fungi (5.13), and *Y. lipolytica* yeast (5.18) with a fermentation time of 4 days (Table 1). The pH value of FPW decreased on day 4 due to the accumulation of organic acids as by‐products produced during the fermentation process (Sulistijowati et al. 2015; Karimi et al. 2018). This event arises due to a metabolic process that produces pyruvate during fermentation and produces acidic by‐products, such as butyric acid, acetic acid, acetone, acetaldehyde, and alcohol (Toe et al. 2019). This opinion is in line with what was stated by (Gaviria Gaviria et al. 2021), that the pH value of a fermentation product is closely related to metabolite compounds in the form of organic acids and fuses to suppress pathogenic bacteria. The lipopeptides in the secondary metabolite *P. aeruginosa* can potentially work as a new type of antibacterial agent (Mok et al. 2019). It is reported that *P. aeruginosa* bacteria can lower the pH value of substrates through the mechanism of lactate dehydrogenase formation (Florek et al. 2024). Low pH conditions inhibit the growth of putrefactive and pathogenic bacteria that are not resistant to acidic conditions, but nutritional and organoleptic values can be maintained (Ishiai et al. 2023).

On the basis of Table 1, the fermentation process results in temperature changes in the FPW media. The highest temperature of FPW was found in the use of *P. aeruginosa* (34.97°C) bacterial species, *R. microsporus* (32.75°C) fungi, and *Y. lipolytica* (34.45°C) yeast with a fermentation time of 4 days. An increase in temperature indicates the occurrence of optimal substrate breakdown followed by the release of energy in the form of heat, CO_2_, and water vapor, resulting in heat (increased fermentation temperature) and accelerated microbial growth (Liu et al. 2022). At warm temperatures (32.75°C–34.97°C), it can be an excellent microbial growing medium characterized by the highest number of microbial colonies. After reaching the peak (at the fermentation time on day 4), the fermentation temperature decreases again (on day 8). This decrease occurs due to microbial activity to decompose organic matter and a decrease in the nitrogen content available in the substrate (Kusmiati et al. 2024).

On the basis of Table 1, it can be seen that FPW using one microbe, the average TPC value increased on day 4 and decreased again on day 8. Microbial species do not affect the TPC value but are more affected by the fermentation time. Along with the length of fermentation time, the TPC value increases until the fourth day, then decreases again on the eighth day. The highest TPC values were found in the use of *P. aeruginosa* bacteria (156 × 10× ^9^ cfu/mL), *R. microsporus* fungi (146 × 10× ^9^ cfu/mL), and *Y. lipolytica* yeast (93 × 10× ^9^ cfu/mL) with a fermentation time of 4 days. The total microbial colonies on FPW increased as microbial growth was aided by environmental suitability and nutrient availability, both regulated by a markedly elevated fermentation medium temperature on day 4. An increase in the number of microbial colonies *of P. aeruginosa* inoculated as fermenter agents is beneficial in the inhibition activity of other bacteria. Bacteriocins (Pyocin) produced by local strains *of P. aeruginosa* QDD1 have high inhibitory activity against Gram‐positive bacteria, including *S. aureus*, *Bacillus cereus*, *B. thuringiensis*, and *B. subtilis* (Doshi et al. 2022).

The moisture content increases on day 4 and then decreases again on day 8. The moisture content range with a single microbe is 58.53%–73.57%. The highest moisture content was found in the use of *P. aeruginosa* bacteria (73.57%), *R. microsporus* fungi (73.15%), and *Y. lipolytica* yeast (72.83%) with a fermentation time of 4 days. Microbes can grow optimally at a water content of 60%–80% (Abun et al. 2021). According to Bidhan et al. (2014), the fermentation process does not cause a significant change in water content. The maximum water content obtained in each type of bacteria is the same as the initial fermentation condition. However, according to the study's results (Ha et al. 2017), the fermentation process can reduce the moisture content. Another factor contributing to the decrease in FPW moisture content is resistance caused by microbial activity (Ishiai et al. 2023; Roselló‐Soto et al. 2019). The higher the temperature and fermentation time, the lower the moisture content of the material due to water evaporation (Vera Zambrano et al. 2019).

#### 4.1.2 Nutrient Content of FPW From Single Microbes

Table 2 shows that FPW products with single microbes have the highest crude protein levels on day 2 and decrease on days 4 and 8. The use of microbial type and fermentation time affect the crude protein content of FPW. The bacterial species *P. aeruginosa*, fungi *R. microsporus*, and yeast *Y. lipolytica*, with a fermentation time of 2 days, produced the highest protein levels, which were 40.80%, 39.77%, and 38.69%, respectively. The longer the fermentation lasts, the more organic components and dry matter are overhauled, so the fermentation time must be limited in producing the expected nutrient content (Marti‐Quijal et al. 2020). The crude protein content on the fourth and eighth days decreased due to the growth rate of microbes, meaning that the metabolic concentration continued to increase until it reached a specific limit, and then there was a decrease (M. Mirzah et al. 2020). The reduction in protein levels occurs due to achieving stable conditions for microbial growth. When microbial growth has reached the stationary phase, the growth rate decreases, which results in a reduced nutrient supply and the accumulation of metabolites that inhibit growth so that the growth rate can continue to decline until it reaches the death phase (M. H. Mirzah 2016). Optimal fermentation time and a balance of nutrients between the substrate and enzymes from microbes are required to obtain high protein levels. The decrease in microbial enzyme activity can be caused by decreased substrate availability (Marti‐Quijal et al. 2020). In addition, according to Lim et al. (2019), the total amount of microbes and metabolite products such as ethanol, carbon dioxide, nitrogen oxides, and antimicrobial substances from proteins or peptides that can inhibit proteolytic bacteria. Therefore, microbial fermentation with a fermentation time of 2 days is the optimal condition for *P. aeruginosa* bacteria, *R. microsporus* fungi, and *Y. lipolitica* yeast to produce the highest protein.

The fat content increased (Table 2) with fermentation time, which ranged from 36.50% to 53.48%, with the lowest crude fat found in the use of *P. aeruginosa* bacteria (36.50%), *R. microsporus* fungi (38.39%), and *Y. lipolytica* yeast (39.32%) at the 2‐day fermentation time. The increase in fat content during fermentation is due to the mass of microbial cells that multiply on the medium (Chen and Liu 2021). This is in line with research that the increase in fat content during the fermentation process is caused by microbial activity breaking down organic matter, such as carbohydrates, into more straightforward bonds to be used in the breeding process. In the fermentation process, there is microbial activity that produces fatty acids high enough to increase fat levels. Fermentation of pangasius waste with *A. niger* fungi produced the highest fat content (53.48%) on the eighth day. This is because the longer the fermentation process, the more carbohydrates are broken into fatty acids, thus increasing fat levels (Shabani et al. 2019). *P. aeruginosa* is more effective in reducing fat at 2 days of fermentation. This shows that in addition to the activity in the fat medium (lipophilic), it can also decompose fat (lipoly) better (Florek et al. 2024).

Table 2 shows that FPW using one type of microbe, crude fiber levels on days 4 and 8, tend to decrease. The lowest crude fiber was found in the use of *P. aeruginosa* bacteria (2.30%), *R. microsporus* fungi (2.50%), and *Y. lipolytica* yeast (2.41%) with a fermentation time of 8 days. Hydrolysis of crude fiber by acids produced by *P. aeruginosa*, *R. microsporus*, and *Y. lipolytica* results in their conversion into simpler molecules, which results in a decrease in crude fiber content during fermentation. The enzyme activity produced by microbes during fermentation reduces the crude fiber content in the fermentation of pangasius waste (Kusmiati et al. 2024).

The moisture content of the dried product (Table 2) tends to increase on the fourth–eighth day. The moisture content range of FPW products with single microbes (after drying) is 8.33%–13.87%. The lowest moisture content was found in the use of *P. aeruginosa* bacteria (8.33%), *R. microsporus* fungi (8.97%), and *Y. lipolytica* yeast (10.15%) with a fermentation time of 2 days. In line with the results of research (Ha et al. 2017), the fermentation process can reduce the moisture content. Another factor contributing to the decrease in FPW moisture content is resistance caused by microbial activity (Ishiai et al. 2023; Roselló‐Soto et al. 2019). The higher the temperature and fermentation time, the lower the moisture content of the material due to water evaporation (Vera Zambrano et al. 2019).

On the basis of the results of macroscopic observations and the chemical quality of FPW products, the best single types of microbes were obtained, namely, *P. aeruginosa* bacteria, *R. microsporus* fungi, and *Y. lipolytica* yeast. The selected microbes are combined in the pangasius waste fermentation process to get the best products from FPW to be used as feed ingredients in compiling poultry feed formulas.

### 4.2 Effect of the Use of Microbial Consortium on the Physical and Chemical Quality of FPW

#### 4.2.1 Macroscopic Observations

Using microbial consortiums *P. aeruginosa*, *R. microsporus*, and *Y. lipolytica* (Pa + Rm + Yl) at a consortium dose of 15% and a fermentation period of 4 days (Table 3) was the best treatment for macroscopic observations. The activity of the microbial consortium in the fermentation process significantly decreased the pH value of FPW, with the lowest pH in the Pa + Rm consortium (5.55), the Pa + Yl consortium (5.58), the Rm + Yl consortium (5.51), and the Pa + Rm + Yl consortium (4.88). A consortium of three types of microbes (Pa + Rm + Yl) produced a lower pH value of FPW (4.88) compared with single‐microbial fermentation (5.1). This suggests that fermentation with a consortium of microbes is more effective in lowering the pH value than single microbes. In line with the study's results (Gaviria Gaviria et al. 2021), the pH value of fermented products is closely related to metabolite compounds in the form of organic acids and fuses to suppress pathogenic bacteria. The microbes *P. aeruginosa*, *R. microsporus*, and *Y. lipolytica* work synergistically as antibacterial agents (Mok et al. 2019). It is reported that such microbes can lower the pH of the substrate through the formation mechanism of lactate dehydrogenase (Florek et al. 2024). Low pH conditions inhibit the growth of putrefactive and pathogenic bacteria that are not resistant to acidic conditions, so that the nutritional and organoleptic value of fermented products can be maintained (Ishiai et al. 2023).

The fermentation temperature is related to the level of microbial activity; the higher the temperature, the higher the microbial activity in decomposing organic matter (Marti‐Quijal et al. 2020). The highest temperature in FPW with microbial consortium (Table 3), namely, Pa + Rm consortium at 33.30°C, Pa + Yl consortium at 33.13°C, Rm + Yl consortium at 33.73°C, and Pa + Rm + Yl consortium at 35.40°C, at a consortium dose of 15% and a fermentation period of 4 days. The temperature of the FPW substrate resulting from a consortium of three types of microbes (Pa + Rm + Yl) was slightly larger (35.40°C) than the result of a single microbe (34.97°C). An increase in temperature indicates the occurrence of optimal substrate breakdown followed by the release of energy in the form of heat, CO_2_, and water vapor, increasing fermentation temperature, and acceleration of microbial growth (Liu et al. 2022). This gives the idea that the consortium of three microbes works synergistically, which is characterized by an increase in the temperature of the fermentation substrate.

The highest moisture content was found in the microbial consortium Pa + Rm + Yl (consortium dose 15%, 4 days). According to Vera Zambrano et al. (2019), the high moisture content in the fermentation substrate can affect the characteristics of the material, where the particles in the material become more significant, so that the texture becomes very soft. The ideal moisture content of fermentation varies between 72.4% and 75%, depending on the product, substrate, and organism used (van't Land et al. 2017). Fish waste has a very high moisture, so the semiliquid substrate fermentation method is used and affects the moisture content of the resulting product. The difference in moisture content before and after fermentation is caused by microbial activity during the fermentation process and can increase the moisture content of the material due to the metabolic process (Shintani 2019). The fermentation process that produces water indicates the continuity of the fermentation process (cell metabolism occurs). If the moisture content is below the critical value, microbial activity decreases and becomes inactive, whereas if the moisture content is too high, the movement of air inside the substrate is inhibited. Solid fermentation produces better products compared with liquid fermentation. In SSF, microbes grow in solid material without free water. The goal of SSF is to bring the cultivated microbes to interact strongly with the water‐insoluble substrate and to achieve the highest concentration of nutrients from the substrate (Yafetto 2022). In this study, the fermentation results of the microbial consortium FPW can dry perfectly, and the moisture content of the flour produced is lower, so it is suitable for use as an ingredient in animal feed (poultry).

The fermentation process, in addition to being influenced by nutritional factors and time, is also influenced by incubation temperature factors, so that it can affect the work of microbiological activities (M. Mirzah et al. 2020). The highest amount of TPC in the microbial consortium (Table 3), namely, in the Pa + Rm consortium of 131.37 × 10× ^9^ cfu/mL, the Pa + Yl consortium of 139.53 × 10× ^9^ cfu/mL, the Rm + Yl consortium of 74.78 × 10× ^9^ cfu/mL, and the Pa + Rm + Yl consortium of 216 × 10× ^9^ cfu/mL, at a consortium dose of 15% with a fermentation time of 4 days. The total microbial colonies in FPW increased as microbial growth was aided by environmental suitability and nutrient availability. An increasing number of microbial colonies of *P. aeruginosa*, *R. microsporus*, and *Y. lipolytica* inoculated as fermenter agents synergize in their growth and benefit the inhibitory activity of other bacteria (Haetami et al. 2023). In the single‐microbial method, *P. aeruginosa*, with a fermentation time of 4 days, the TPC value was 156 × 10× ^9^ cfu/mL (Table 1), while in the microbial consortium method Pa + Rm + Yl (dose 15%, for 4 days), it was 216 × 10× ^9^ cfu/mL (Table 3). This shows that the consortium method in FPW is more effective than single microbes.

#### 4.2.2 Nutrient Content of FPW From Consortium Microbes

On the basis of Table 4, it can be seen that in FPW using a microbial consortium, the average protein content increases with fermentation time, but some decrease with fermentation time. The highest protein content was in the Pa + Rm consortium of 49.71%, the Pa + Yl consortium of 50.62%, the Rm + Yl consortium of 50.25%, and the Pa + Rm + Yl consortium of 52.02%, at a consortium dose of 15% and a fermentation period of 4 days. A microbial consortium is a collection of microorganisms that work together to degrade organic molecules and are more efficient than single microbes (van't Land et al. 2017; Mamma 2020). The increase in crude protein levels is due to the growth and expansion of microorganisms during the fermentation process, thereby increasing the protein‐rich microbial community. The increase in unicellular protein microbial colonies during the fermentation process indirectly increases the crude protein levels (Shabani et al. 2021). The correct dose of the inoculum results in rapid growth and the right fermentation time. It concerns the balance of nutrients between the substrate and microbial enzymes. As a result, after 2 days of fermentation (day 4 to be precise) with an inoculum dose of 15%, the microbial consortium Pa + Rm + Yl produced the highest protein levels. The crude protein content of FPW, produced by the microbial consortium, is higher than that of single microbes. The protein content of FPW produced by a single microbe, with the highest value of 40.80% (*P. aeruginosa*), was still lower than the protein content of FPW produced by the microbial consortium Pa + Rm + Yl (52.02%).

Most fat levels decrease as fermentation time increases (Table 4). The fat content of FPW was evaluated using an extraction process using hexane organic solvents to extract or break down fats (Guo et al. 2022). Fermentation can increase or decrease the level of fat produced. In the fermentation process, there is microbial activity that produces fatty acids that are high enough to increase fat levels. Biomass values indicate that at pH 6.0, *Y. lipolytica* exhibits the same growth value compared with pH 7.0, but its growth is inhibited at pH 8.0, with biomass values between 0.87 and 1.7 g of dry cell weight *L* − 1 (Bilal et al. 2021). The fermentation process with the microbial consortium produced the lowest average fat content, namely, in the Pa + Rm consortium of 38.26%, the Pa + Yl consortium of 37.28%, the Rm + Yl consortium of 36.62%, and the Pa + Rm + Yl consortium of 29.24%, at a consortium dose of 15% and a fermentation period of 4 days (except for the Pa + Rm consortium with a fermentation time of 8 days). The crude fat content of FPW from the Pa + Rm + Yl microbial consortium was much lower than that of a single microbial (36.50%, *P. aeruginosa*). The activity of microbes can degrade other components, including proteins and carbohydrates. The breakdown can produce chemicals that are converted by microorganisms into fat. Lipophilic microbes effectively break down fats during fermentation, resulting in a decrease in fat levels. Lipophilic organisms can grow in fat media, generally as microphilic compounds (Johannah et al. 2018). The term lipolytic microbes are microorganisms that produce the lipase enzyme needed to break down fats or oils (Bilal et al. 2021). The lipase enzyme catalyzes the hydrolysis of ester bonds in triglycerides, producing free fatty acids and glycerol (Wang et al. 2020). This is in line with the fact that lipase breaks down large amounts of fat into fatty acids (both saturated and unsaturated). Microbes that can be cultured on high‐fat media (lipophilic) effectively use fat as a source of nutrients for their metabolism (Zhou et al. 2020).

The fermentation of pangasius waste, by utilizing a consortium of microbes, generally reduces the content of crude fiber. The lowest crude fiber content in FPW by the microbial consortium was in the Pa + Rm consortium of 2.35%, the Pa + Yl consortium of 2.41%, the Rm + Yl consortium of 2.42%, and the Pa + Rm + Yl consortium of 2.19%, at a consortium dose of 15% and a fermentation period of 4 days (except for Pa + Rm for 8 days). The fermentation technique reduces the concentration of crude fiber in pangasius waste, increases protein solubility, improves amino acid profiles, increases the availability of vitamins, minimizes antinutrient compounds, and increases the palatability of feed ingredients (Sukma et al. 2018). According to Ramadhani et al. (2020), fermentation time can affect the crude fiber content due to microbial proliferation, resulting in the multiplication of cellulose‐containing hyphae. As a result, the crude fiber content will grow as the fermentation process continues. Furthermore, increasing inoculum dosage and incubation duration can increase crude fiber content and enzyme production.

The moisture content in the sample is a crucial parameter that shows the quality of the feed material produced. This quality metric is greatly influenced by environmental factors and the nature of the feed material itself. The moisture content of the feed is affected by factors, such as the type of material, ambient temperature, and humidity level (Tan et al. 2019). Generally, lower moisture content in feed ingredients indicates better quality in the feed ingredients produced. Samples with low moisture content can prevent the growth of damaging microorganisms. This is based on the statement (Shabani et al. 2021) that the moisture content of a feed ingredient can affect its shelf life because microbial activity is increasingly inhibited along with decreasing moisture content. Microbial activity accelerates the process of releasing CO_2_ and H_2_O and facilitates the drying process. This follows the opinion (Senz et al. 2019; Abun et al. 2025) that the lower the moisture content of an ingredient, the longer the shelf life of the material, which describes the quality of the feed. Low moisture content means that the sample has high absorbency. Low water content describes low intracellular fluid, which means the sample material is not saturated and provides an opportunity to absorb water (Abun et al. 2025). The moisture content of FPW products (after drying) with a consortium of *P. aeruginosa*, *R. microsporus*, and *Y. lipolytica* microbes (Table 4), the result is below 10%, which indicates that it is suitable as a feed material for poultry.

### 4.3 Potential of FPW Products as Feed Ingredients

Using pangasius waste in a biological way (fermentation) is an effort toward zero waste and a green economy, and its use can be increased as a raw material for feed for animals (especially poultry). Providing rations containing FPW feed ingredients resulting from biological processes is expected to increase positive responses to poultry performance, considering that fermented products have relatively good physical and chemical properties compared with the original ingredients. The study results (Tables 1, 2, 3, 4) show that selected microbial species were obtained for each type of microbe. This microbial selection is to obtain microbes that are suitable to work in pangasius waste media to prevent continued damage from fish waste that is susceptible to high fatty oxidative damage and protein autolysis. Damage occurs through three mechanisms: contaminated with spoilage microorganisms, biochemical activity in waste that can degrade quality, and physical changes due to poor handling (van't Land et al. 2017). The results of this study provide clarity on the fact that fermented products can be used as feed ingredients. In the drying process of high moisture content, when the moisture content of the sample is close to equilibrium, the drying rate decreases (Zapata‐Campos et al. 2020). This phenomenon is obtained from the fermentation process, which can increase the temperature. Drying dried products (oven method 82°C, day 2). In this study, the drying temperature affects the amount of water that can evaporate from the material without damaging the nutrient content. Higher drying temperatures can accelerate the heat transfer and evaporation rate of water from the drying process (Erensoy et al. 2020). The reduced water content in feed ingredients (FPW) is due to changes in texture and pores. The water content of FPW from the microbial consortium ranges from 8% to 11% (Table 4), and has met the ideal water content to be used as a feed ingredient is 10%. Feed ingredients with high water content create an excellent habitat for microbial growth due to the abundance of free water. Opinion (Tapía et al. 2020) that water decomposes more when water‐containing molecules are stored for a long time, as long as the development of bacteria continues. The main factors determining the quality of stored feed ingredients are temperature, humidity, and atmospheric composition (Lakmini et al. 2022).

On the basis of the information in Tables 1, 2, 3, 4, FPW products with consortium microbes are more likely to be used as feed materials, when compared with single‐microbial fermentation products. On the basis of the results of observation of physical and chemical properties, the treatment of a consortium of three microbial jebis (Pa + Rm + Yl) (dose 15%, day 4) can be used as a feed ingredient for poultry (meeting the nutritional needs of poultry).

## 5 Conclusion

On the basis of the results of the study, it was concluded that the fermentation of pangasius waste using three types of microbes (*P. aeruginosa* bacteria, *R. microsporus* fungi, and *Yarrowia lipolytica* yeast) in the consortium at a dose of 15% and a fermentation time of 4 days, produced the best physical and chemical properties of fermented products. Pangasius waste fermentation products (FPW) can be used as feed ingredients as a source of protein for poultry (crude protein content of 52.02%, crude fat 29.24%, and crude fiber 2.19%, with a moisture content of 8.82%).

## Author Contributions

**Abun Abun:** conceptualization, funding acquisition, writing – original draft, methodology, writing – review and editing, validation, formal analysis, supervision, resources. **Kiki Haetami:** investigation, writing – original draft, visualization, writing – review and editing, software, project administration, data curation. **Denny Rusmana:** investigation, writing – original draft, validation, visualization, writing – review and editing, software, formal analysis, data curation. **Rahmad Fany Ramdhan:** software, data curation, formal analysis, supervision, visualization, project administration, writing – original draft, writing – review and editing.

## Ethics Statement

The authors have nothing to report.

## Conflicts of Interest

The authors declare no conflicts of interest.

## Data Availability

Data sharing does not apply to this article as no data sets were generated or analyzed during the current study.
